# Prevalence and selected predictors of vitamin D deficiency among children and adolescents attending primary health care centers: A cross-sectional record-based study, Qatar

**DOI:** 10.5339/qmj.2025.112

**Published:** 2025-12-11

**Authors:** Hanan Khudadad, Ahmed Alnuaimi, Shajitha Thekke Veettil, Abduljaleel Abdullatif Zainel

**Affiliations:** 1 Clinical Research Department, Directorate of Clinical Affairs, Primary Health Care Corporation, Doha, Qatar *Email: sveettil@phcc.gov.qa

**Keywords:** Vitamin D, deficiency, children, adolescents, primary care, Qatar

## Abstract

**Background:**

Vitamin D deficiency is increasingly recognized as a significant public health issue, with far-reaching effects on bone health and overall well-being, especially among children and adolescents.

**Aim/Objectives:** This cross-sectional, record-based study aimed to assess the prevalence and predictors of vitamin D deficiency in individuals under 18 years old who attended Primary Health Care Corporation (PHCC) centers in Qatar between 2018 and 2019.

**Methodology:** Data were extracted from 48,947 electronic medical records, each containing at least one valid serum vitamin D test result. Severe deficiency was defined as serum vitamin D levels <10 ng/ml. Participants undergoing vitamin D therapy were excluded from the prevalence analysis.

**Results:**

Findings revealed that infants (under 1 year) and children aged 1–4 years had the lowest rates of severe deficiency at 3.8% and 3.4% respectively. The prevalence increased with age, reaching 40% among adolescents (10–17 years). Females had significantly higher rates of severe deficiency (30.4%) compared to males (15.3%).

Multivariate logistic regression identified age, sex, and nationality as significant predictors. Adolescents were 17 times more likely to have a severe deficiency compared to children under 5 years. Females had a 2.4-fold increased risk, and individuals from Southern Asia had a 5.7-fold higher risk compared to other nationalities.

**Conclusion:**

This study highlights a high prevalence of vitamin D deficiency among adolescents in Qatar, particularly among females and certain ethnic groups. To address this emerging health issue in the pediatric population, targeted interventions, such as awareness campaigns, supplementation programs, and policy-level strategies, are essential.

## 1. INTRODUCTION

Vitamin D is a fat-soluble vitamin essential for various bodily functions and acts as a prohormone. There are two primary forms: vitamin D2 (ergocalciferol) and vitamin D3 (cholecalciferol).^1^ Uniquely, our bodies can synthesize vitamin D when exposed to ultraviolet B rays from the sun. Additionally, it can be obtained from dietary sources, such as fatty fish (e.g., salmon, mackerel, and tuna), fortified foods (e.g., fortified milk and cereals), and supplements.^2^

Vitamin D is involved in numerous bodily functions, including calcium and phosphorus homeostasis, maintaining bone health, supporting the immune system, cell growth and differentiation, and overall health.^3^ Maintaining adequate levels of vitamin D is crucial, as deficiency can lead to health problems like rickets in children and osteomalacia in both children and adults.^4,5^ Despite the availability of sunlight as a natural source, a significant portion of the world’s population suffers from insufficient levels of this essential nutrient, making vitamin D deficiency and insufficiency recognized global health problems.^6^

The widespread issue of vitamin D deficiency can be attributed to several factors. Limited sunlight exposure, influenced by climate, lifestyle, cultural practices, or living in regions with reduced sun exposure, is a significant contributor.^7^ Dietary factors also play a role, as many individuals have diets lacking in vitamin D-rich sources like fatty fish, egg yolks, and fortified dairy products. Additionally, certain medical conditions and gastrointestinal disorders can hinder the absorption of vitamin D from food, supplements, or sunlight, increasing the risk of deficiency. Addressing these factors is essential to combat vitamin D deficiency and promote overall health.^8^

Infants and young children are among the groups at higher risk of vitamin D deficiency.^9^ This age group is particularly vulnerable to the long-term consequences of prolonged vitamin D deficiency, including cardiovascular disease, diabetes, and autoimmune diseases.^10^ Estimates suggest that approximately 30% of children worldwide are vitamin D-deficient. There is no universal consensus on the biochemical definition of vitamin D deficiency. Expert groups and bodies have varying recommendations for optimal vitamin D levels. The US National Academy of Medicine recommends a minimum serum 25-hydroxyvitamin D [25(OH)D] concentration of 50 nmol/L (equivalent to 20 ng/mL) for adequate bone health.^11^ This recommendation is based on the role of vitamin D in calcium absorption and bone mineralization. On the other hand, the US Endocrine Society suggests a higher target level of at least 75 nmol/L (equivalent to 30 ng/mL) for overall health benefits, including reducing the risk of non-communicable diseases like cardiovascular diseases, diabetes, and autoimmune conditions, as well as certain infectious diseases.^12^ Other countries and medical organizations may also have their own guidelines and recommendations for vitamin D levels. It’s important to note that individual factors, such as age, underlying health conditions, and specific health goals, can influence the optimal vitamin D levels for a person. Furthermore, the interpretation of vitamin D levels should be done in conjunction with other clinical factors and the guidance of healthcare professionals.

In current pediatric practice, severe vitamin D deficiency is defined as a plasma 25(OH)D level of less than 25 nmol/L (10 ng/mL). This threshold is based on the observation that symptomatic vitamin D deficiency in children, such as rickets and hypocalcemia, typically occurs below this level. The Qatar Primary Health Care Corporation (Qatar-PHCC) guidelines align with this approach.^13^

The rationale was emphasized more strongly and explicitly in the last paragraph of the introduction. In Qatar, numerous studies have assessed vitamin D levels in hospital settings or through small-scale surveys. In 2017, a manuscript was published presenting a situational analysis of vitamin D levels in a large sample of adults receiving primary health care services in Qatar. The study revealed a high prevalence rate (14.1%) of severe vitamin D deficiency among adults aged 18 to 65 years. When the threshold for defining prevalence was lowered, the deficiency rate increased to 71.4%, and the insufficiency rate rose to 92.7%.^14^ This study naturally led to the current research, which aims to determine the prevalence of vitamin D deficiency and associated factors among children and adolescents under 18 years old attending primary health care centers in Qatar during 2018 to 2019.

The current study provides a unique opportunity to study the epidemiology of vitamin D deficiency in the vulnerable group of children in the multinational context of Qatar. In this respect, the local context of Qatar can apply to the international context, given the very large sample size studied. This highlights the importance of studying it in different contexts, including rapidly developing countries with diverse populations, such as Qatar. In addition, findings from Qatar are relevant internationally, particularly for regions with similar climates, sociocultural practices, and dietary patterns (e.g., the Middle East, North Africa, and South Asia). This contributes to the global body of evidence and supports international comparisons in pediatric vitamin D deficiency.

### 1.1 Study hypothesis

Key sociodemographic factors such as age, sex, obesity, and nationality group are significant predictors of vitamin D deficiency among children and adolescents in the Qatari population.

Objectives of the study:

- To estimate the prevalence of vitamin D deficiency and severe vitamin D deficiency among children and adolescents (<18 years) attending primary health care centers in Qatar during 2018 to 2019.
- To examine demographic and clinical predictors of vitamin D deficiency, including age, sex, nationality, and obesity status.
- To compare prevalence patterns across different pediatric age groups (infants, young children, school-age children, and adolescents).

## 2. MATERIALS AND METHODS

1. **Study design:** Cross-sectional study.
2. **Study setting:** The PHCC is the largest publicly funded primary care provider in Qatar. At the time of this study, PHCC was running 29 health centers across the country. Every individual with a valid residence permit in Qatar is eligible to utilize the subsidized services provided by PHCC.
3. **Study population:** The electronic medical records (EMR) of 48,947 children and adolescents under 18 years old with valid serum vitamin D test results from 2018 to 2019 were analyzed under 3 groups as preschool age (<5 years), school age (5–9 years), and teenagers (10–17 years). At PHCC, vitamin D tests are conducted as part of routine health checks and for individuals with specific health concerns. The inclusion criteria for this study were as follows: participants under 18 years old (<216 completed months) at the time of the vitamin D test, had a valid test result during the two-year study period (January 1, 2018, to December 31, 2019), and had non-missing values for sex, nationality, body height, and weight. No exclusion criteria or sampling techniques were applied, as this is a population study.
4. **Study variables:** The outcome variable is vitamin D deficiency, defined at three cut-off values: severe deficiency (<10 ng/mL), deficiency (10–19.9 ng/mL), and insufficiency (20–29.9 ng/mL).
  The independent (exposure) variables include age, sex, body mass index (BMI), and nationality.
  The World Health Organization (WHO) growth standards were used to calculate BMI-for-age Z-scores. BMI Z-score cut-points of < −2.0 and > +2.0 were used to define the wasted and overweight/obese categories for non-adults (children and adolescents).^15^ Calculations of anthropometric indices were done using the WHO Anthro software.^16^
  The nationality categories were defined according to the updated list of United Nations-registered countries, with the coding system described in the supplementary files part of the manuscript.
5. **Data Collection:** The PHCC family physicians are authorized to request vitamin D serum for any patient under their care. Blood samples are collected by trained phlebotomists using standardized procedures and analyzed locally in the health center laboratory. The test results are recorded and stored in PHCC’s EMR system (CERNER). Data was extracted for 2 years from January 1, 2018, to December 31, 2019. The PHCC CERNER system uses SNOMED codes, which provide standardized medical terms for clinical documentation and reporting. The Business Health Intelligence (BHI) department provided the list of variables using custom filters that meet the inclusion criteria for the defined study population. If multiple serum tests were conducted for the same individual during the study period, the most recent result was used.
6. **Data analysis:** The Statistical Package of Social Sciences (IBMSPSS Ver 27) was used for data analysis. Data cleaning involved logical checks for consistency of related variables and range checks for dates within the specified study period. Since vitamin D replacement therapy can affect serum vitamin D levels, the data were divided into two groups: those treated with vitamin D replacement therapy before the test date and those untreated. Descriptive statistics summarized the demographic characteristics of the study population, including age, gender, and nationality. Proportions provided a clear overview of the participants’ profiles. The association between the explanatory variables and the probability of vitamin D deficiency was tested at the bivariate level using the odds ratio (OR) for the effect size and the chi-square test for statistical significance. A multivariate model (Multiple Logistic Regression) was used to investigate the impact of different factors on the likelihood of vitamin D deficiency, identifying significant predictors and their relative contributions. P-value <0.05 was considered statistically significant. The extremely large sample size allowed for an overpowered analysis, with a study power of 96% for an alpha of 0.05 and a sample size of 42,000 (the untreated group) to detect a minute difference of 0.01 in an adjusted OR (multiple logistic regression) as small as 1.2. The study power reached 99.9% for any adjusted OR >1.3.^17^
7. **Quality control measures:** In the preparation phase of the study, an extensive literature review was conducted. The study authors, in collaboration with the BHI department, were responsible for data collection and extraction. The data extraction filters used by the BHI were double-checked and tested for performance. Blood collection and laboratory analysis followed PHCC’s standard operating procedures. Serum vitamin D was tested using the Abbott ARCHITECT i1000SR IMMUNO analyzer, which performs automated immunoassay tests utilizing CMIA (chemiluminescent microparticle assay) detection technology. Laboratory internal quality control was routinely conducted at the beginning of the morning shift and assessed using Westgard rules and Levey-Jennings plots once daily. External quality control followed the RIQAS (Randox International Quality Assessment Scheme) protocol.
8. **Ethical Consideration:** The study presented minimal risk of harm to its human participants. The data extracted from health records were anonymized. None of the study participants’ personal information was revealed to the research team. Overall, the study was conducted with integrity according to generally accepted ethical principles and was approved by the PHCC’s Institutional Review Board (IRB).
9. **Patient and Public Involvement:** There was no direct intervention/interaction with study participants. Patients’ priorities, experiences, and preferences were not gathered, nor were they involved in designing the study. There are no plans to disseminate results to the study participants directly, as they were anonymized.

## 3. RESULTS

The results presented were based on the analysis of data extracted from 48,947 EMRs of children under 18 years old with at least one valid serum vitamin D test result. The sociodemographic characteristics of the participants are presented in Table 1.

Only 6562 of the total sample received one or multiple forms of vitamin D replacement therapy at any point in time during the 2-year study period before the serum testing available in the EMR. This group of individuals had a lower prevalence of severe vitamin D deficiency (16%) compared to those with no evidence of prior therapy (23.5%). Consequently, this group was excluded from the remaining analyses included in this chapter, as shown in Table 2.

For the study participants with no history of vitamin D replacement therapy, the crosstabulation showed that children aged one to four years and infants under one year old had the lowest rate of vitamin D deficiency (3.4% and 3.8%, respectively). This rate increases with age, reaching the highest value (40%) among teenagers (10–17 years old). Males had a much lower rate of severe vitamin D deficiency (15.3%) compared to females (30.4%). Additionally, nationalities from Western Asia and Southern Asia had the highest rates of severe vitamin D deficiency (27.9% and 24.4%, respectively), while those from Northern America and Northern Europe had the lowest rates (7.1% and 7.3%, respectively). Furthermore, BMI was positively correlated with the probability of severe vitamin D deficiency. The overweight/obese group had the highest prevalence of severe vitamin D deficiency (31%), while the wasted group had the lowest rate (14.6%; Table 3).

Multivariate modelling was used to predict severe vitamin D deficiency based on age, gender, nationality, and obesity. As shown in Table 4, all predictors had a significant association with the risk of severe vitamin D deficiency. Teenagers (10–17 years old) were 17 times more likely to have severe vitamin D deficiency compared to preschool-aged children (<5 years old), after adjusting for the possible confounding effect of other explanatory variables included in the model. Females increased the risk by 2.4 times compared to males, and Southern Asia nationality by 5.7 times compared to other nationalities. The overweight/obese group had a 2.6 times higher risk compared to the wasted group. The model was statistically significant and accurately predicted severe vitamin D deficiency status with an overall accuracy of 77.8%. All the calculated risk estimates in multivariate modelling were not that different from those calculated in the bivariate model discussed in Table 3. The 95% confidence interval for the risk estimates showed a narrow error range due to the extremely large sample size.

## 4. DISCUSSION

This cross-sectional review of health records explored the prevalence of vitamin D deficiency and selected associated factors among children and adolescents under 18 years old in Qatar’s largest publicly funded primary health care provider. This study aimed to complement previous research on vitamin D deficiency among adults in Qatar.^14^ The strength of this study lies in its use of population data to assess the magnitude of this health issue.

### 4.1 Magnitude

Between 2009 and 2022, Qatar saw a significant reduction in the prevalence of vitamin D deficiency, nearly halving the rates.^18^ This decline can be attributed to the effective implementation of guidelines for addressing and managing vitamin D deficiency among children within primary healthcare centers. This development may explain the notable prevalence of insufficient vitamin D levels observed in the primary healthcare population.

### 4.2 Nationality

The prevalence of childhood vitamin D deficiency and insufficiency varies across studies worldwide. Research conducted in Asian countries showed a notably higher occurrence of vitamin D deficiency compared to studies in Europe, North America, and New Zealand.^19–27^ Several explanations have been proposed for these geographic differences. Individuals with darker skin tones, as indicated by factors such as race, ethnicity, or skin pigmentation, are at a higher risk for insufficient vitamin D levels.^19,21,24,27^ In regions where vitamin D is fortified in milk and/or juice, a lack of consumption of these fortified products is linked to an elevated likelihood of deficiency. This observation underscores the role of fortification as an effective strategy to enhance the vitamin D status among children.^19,21,24^ The substantial prevalence of vitamin D deficiency observed in the current study corresponds to similar outcomes documented within diverse subgroups across the Middle Eastern region, as well as in prior investigations conducted in Qatar. A systematic review specifically focused on Qatar demonstrated a very high prevalence (>80%) of vitamin D insufficiency (<30 ng/mL or 75 nmol/L).^18^ Another comprehensive systematic review highlighted that severe vitamin D deficiency (<10 ng/mL) was most prevalent in South Asia and the Middle East.^28^ These two nationalities had an exceptionally higher prevalence of severe vitamin D deficiency in the current study. An interesting finding that highlights the role of nationality/ethnicity in vitamin D status comes from studying ethnic minority groups with origins from Arab countries, residing in northern Europe, the United States, and Australia. For instance, over 80% of Moroccan women immigrants in the Netherlands suffered from severe deficiency, a prevalence rate similar to that reported among veiled women in Australia.^29^ The association between nationality and severe deficiency status is unlikely to be caused by the type of clothing in childhood. Instead, it points to a probable role of culturally acceptable diet by these nationality groups.

### 4.3 Age

The current study highlights a sizable prevalence of vitamin D deficiency among the pediatric and adolescent population. Notably, adolescents aged 10 to 17 years exhibited the highest rates of severe deficiency, followed by children aged 5 to 9 years, while those under five years old showed a much lower rate. A 2009 study by Bener et al. among Qatari youth aged 0 to 16 years reached similar conclusions.^30^ This positive age trend has also been observed in Western populations. Data from Public Health England revealed that 20% of boys and 24% of girls aged 11 to 18 years had 25(OH)D levels below 25 nmol/L.^31^ Interestingly, despite the susceptibility of infants to conditions like vitamin D-related rickets worldwide, many population-based studies in higher-resource nations show lower vitamin D levels among adolescents compared to toddlers.^21,22,31^ This pattern contrasts with findings in lower-resource countries, where a higher prevalence of vitamin D deficiency is observed among infants and toddlers, particularly those who are breastfed.^26^

### 4.4 Sex

In the present study, a more pronounced occurrence of severe vitamin D deficiency was observed among females (30.4%) compared to males (15.3%). Similarly, a study encompassing participants aged over 18 years in Jordan found that low vitamin D status was more prevalent in females. The traditional dress code followed by females in the Muslim culture of Qatar was independently linked to low vitamin D status. Specifically, women wearing 'Hijab' (adjusted OR = 1.7; P = 0.004) or “Niqab” (adjusted OR = 1.5; P = 0.061) exhibited a heightened vulnerability to low vitamin D status compared to those adopting Western attire.^32^ Additionally, the hot weather in Qatar for most of the year discourages outdoor activities and sun exposure.

### 4.5 Obesity

The potential link between obesity and low vitamin D levels observed in this study may be due to reduced vitamin D bioavailability from both skin and dietary sources among individuals with obesity.^33^

The current PHCC guideline “The Recognition and Management of Vitamin D Deficiency” states that screening for vitamin D deficiency is recommended for individuals at risk, labelling the population-level screening as not recommended.^34^ The current study showed that almost three-quarters of children and adolescents are either deficient or severely deficient in vitamin D. This may be used as evidence to recommend population-level screening in future versions of the guideline. One can also consider lowering the threshold for recommending vitamin D supplementation.

### 4.6 Specific recommendations

Based on national and international evidence, several strategies can help address vitamin D deficiency in children and adolescents.^34^ First, targeted screening of high-risk groups is recommended as a cost-effective approach for early detection. Teenagers were shown to be the most vulnerable age group for severe vitamin D deficiency status. Second, vitamin D supplementation programs should be strengthened to reduce rickets and improve bone mineralization. Third, food fortification of staples like milk, flour, or cereals has proven effective in other countries and could be adapted for Qatar’s context. Fourth, lifestyle interventions, including safe sunlight exposure campaigns and structured outdoor activities in schools, may enhance natural vitamin D synthesis. Finally, public awareness and education initiatives targeting parents and healthcare providers can improve dietary practices, supplementation adherence, and early recognition of deficiency. Together, these evidence-based measures offer a comprehensive, feasible framework to improve pediatric vitamin D status.

## 5. CONCLUSION

This study highlighted the magnitude and importance of vitamin D deficiency among older children and adolescents attending primary health care centers. Females, teenagers, Western and Southern Asian nationalities were found to be at higher risk of severe deficiency.

By implementing comprehensive strategies that encompass education, policy changes, and healthcare provider involvement, we can work towards reducing the prevalence of vitamin D deficiency and ultimately enhance the overall health and well-being of the younger population. Additionally, monitoring of vitamin D levels through blood tests can help assess an individual’s status and guide appropriate interventions, such as sun exposure, dietary changes, or vitamin D supplementation, as needed to maintain healthy levels.^35^

## DATA AVAILABILITY STATEMENT

The datasets generated and/or analyzed during the current study are not publicly available due to the institutional rules and regulations, but are available from the corresponding author on reasonable request.

## ETHICAL APPROVAL

This project has been ethically approved (Ref no: PHCC/DCR/2020/06/052) by the Department of Clinical Research, Research Sub Committee in Primary Health Care Corporation, Doha, Qatar.

## AUTHORS’ CONTRIBUTIONS

HK: Contributed to the conception, design, and data collection, drafted the manuscript, and revised it critically for final content. AA: Contributed the analysis and interpretation of data and revised the manuscript critically for final content. SV: Drafted the manuscript and revised it critically for final content. AZ: Contributed to the conception, design, and drafted the manuscript.

## ACKNOWLEDGMENTS

The authors are extremely grateful to everyone who contributed to this work, especially the HIM Department in PHCC.

## CONFLICT OF INTEREST

The authors declared that the research was conducted in the absence of any commercial or financial relationships that could be construed as a potential conflict of interest.

## Figures and Tables

**Table 1.** Description of study sample.

	*N*	%
**Age group at testing vitamin D (years)**		
Infants (<1)	339	0.7
Less than 5 years (1–4)	8731	17.8
5–9	14273	29.2
Teenagers (10–17)	25604	52.3
Total	48947	100.0
**Gender**		
Female	27991	57.2
Male	20956	42.8
Total	48947	100.0
**Nationality categories**		
Northern Africa	11211	22.9
Sub-Saharan Africa	498	1.0
Northern America	590	1.2
South-Eastern Asia	272	0.6
Southern Asia	6219	12.7
Western Asia	29472	60.2
Northern Europe	394	0.8
Other (miscellaneous) nationality	291	0.6
Total	48947	100.0

**Table 2.** Frequency distribution of the study sample by serum vitamin D categories stratified by vitamin D replacement therapy status at any time before serum testing (during the 2-year study period).

	Vitamin D replacement therapy at any time before the serum testing during the study period			
	Negative		Positive	
Serum vitamin D (ng/mL) categories	*N*	%	*N*	%
Severe deficiency (<10)	9981	23.5	1053	16.0
Deficiency (10–19.9)	20365	48.0	3866	58.9
Insufficiency (20–29.9)	8434	19.9	1121	17.1
Optimum (30+)	3605	8.5	522	8.0
Total	42385	100.0	6562	100.0

**Table 3.** Participants without prior vitamin D therapy before serum testing.

	Sever vitamin D deficiency (<10 ng/mL)*						
	Negative		Positive			Total	
	*N*	%	*N*	%	95% confidence interval	*N*	%
**Age group at testing vitamin D (years)**							
Infants (<1)	326	96.2	13	3.8	(2.2–6.3)	339	100.0
Less than 5 years (1–4)	8427	96.6	299	3.4	(3.1–3.8)	8726	100.0
5–9	11907	86.8	1818	13.2	(12.7–13.8)	13725	100.0
Teenagers (10–17)	11744	59.9	7851	40.1	(39.4–40.8)	19595	100.0
Total	32404	76.5	9981	23.5	(23.1–24)	42385	100.0
**Gender**							
Female	16158	69.6	7056	30.4	(29.8–31)	23214	100.0
Male	16246	84.7	2925	15.3	(14.8–15.8)	19171	100.0
**Nationality categories**							
Northern Africa	8514	85.0	1501	15.0	(14.3–15.7)	10015	100.0
Sub-Saharan Africa	373	84.2	70	15.8	(12.6–19.4)	443	100.0
Northern America	496	92.9	38	7.1	(5.2–9.5)	534	100.0
South-eastern Asia	242	92.0	21	8.0	(5.2–11.7)	263	100.0
Southern Asia	4225	75.6	1366	24.4	(23.3–25.6)	5591	100.0
Western Asia	17964	72.1	6947	27.9	(27.3–28.4)	24911	100.0
Northern Europe	332	92.7	26	7.3	(4.9–10.3)	358	100.0
Other (miscellaneous) nationality	258	95.6	12	4.4	(2.5–7.4)	270	100.0
**Obesity**							
Waisted (< −2 Z)	1941	85.4	333	14.6	(13.2–16.1)	2274	100.0
Acceptable (−2 to 2 Z	24398	78.0	6901	22.0	(21.6–22.5)	31299	100.0
Overweight/obese >2 Z	3853	69.0	1731	31.0	(29.8–32.2)	5584	100.0

*All the calculated *P* values were statistically significant (*P* < 0.001) because of the extremely large sample size.

**Table 4.** Logistic regression with the risk of having severe vitamin D deficiency as the dependent (outcome) variable.

	Bivariate model			Multivariate model		
	OR	95% confidence interval OR	*P* Value	Adjusted OR	95% confidence interval OR	*P* Value
Age group at testing vitamin D (years)						<0.001
School age (5–9 years) compared to preschool age (<5 years)	4. 3	(3.8–4.8)	<0.001	4.0	(3.6–4.6)	<0.001
Teenagers (10–17 years) compared to preschool age (<5 years)	18.8	(16.7–21.1)	<0.001	17.0	(15.1–19.2)	<0.001
Female gender compared to the Male	2.4	(2.3–2.5)	<0.001	2.4	(2.3–2.5)	<0.001
Nationality categories						<0.001
Northern Africa compared to other nationalities (including North America, Northern Europe)	2.4	(1.95–3.0)	<0.001	2.7	(2.1–3.5)	<0.001
Sub-Saharan Africa compared to other nationalities (including North America, Northern Europe)	2.6	(1.9–3.6)	<0.001	3.1	(2.2–4.6)	<0.001
Southern Asia compared to other nationalities (including North America, Northern Europe)	4.4	(3.6–5.5)	<0.001	5.7	(4.4 to 7.3)	<0.001
Western Asia compared to other nationalities (including North America, Northern Europe)	5.3	(4.3–6.5)	<0.001	4.8	(3.8–6.2)	<0.001
Obesity						<0.001
Acceptable (−2 to 2 z) compared to waisted (<−2 z)	1.6	(1.5–1.9)	<0.001	1.0	(0.88–1.1)	0.95 [NS]
Overweight/obese (2 z) compared to waisted (<−2 z)	2.6	(2.3–3.0)	<0.001	1.5	(1.3–1.7)	<0.001
Constant				0.005		<0.001

Overall predictive accuracy = 77.8%; P (model) <0.001.
